# Dual Effect of Serum Amyloid A on the Invasiveness of Glioma Cells

**DOI:** 10.1155/2013/509089

**Published:** 2013-02-25

**Authors:** Franciele Hinterholz Knebel, Renata Chaves Albuquerque, Renato Ramos Massaro, Silvya Stuchi Maria-Engler, Ana Campa

**Affiliations:** Departamento de Análises Clínicas e Toxicológicas, Faculdade de Ciências Farmacêuticas, Universidade de São Paulo, Avenida Prof. Lineu Prestes, 580 Cidade Universitária, 05508-000 São Paulo, SP, Brazil

## Abstract

Evidence sustains a role for the acute-phase protein serum amyloid A (SAA) in carcinogenesis and metastasis, and the protein has been suggested as a marker for tumor progression. Nevertheless, the demonstration of a direct activity of SAA on tumor cells is still incipient. We have investigated the effect of human recombinant SAA (rSAA) on two human glioma cell lines, A172 and T98G. rSAA stimulated the [^3^H]-thymidine incorporation of both lines, but had dual effects on migration and invasiveness which varied according to the cell line. In T98G, the rSAA increased migration and invasion behaviors whereas in A172 it decreased these behaviors. These findings agree with the effect triggered by rSAA on matrix metalloproteinases (MMPs) activities measured in a gelatinolytic assay. rSAA inhibited activity of both MMPs in A172 cells while increasing them in T98G cells. rSAA also affected the production of compounds present in the tumor microenvironment that orchestrate tumor progression, such as IL-8, the production of reactive oxygen species (ROS) and nitric oxide (NO). We also observed that both lines expressed all three of the isoforms of SAA: SAA1, SAA2, and SAA4. These data suggest that some tumor cells are responsive to SAA and, in these cases, SAA may have a role in cancer progression that varies according to the cell type.

## 1. Introduction

Serum amyloid A (SAA) is an acute-phase protein with cytokine-like properties produced predominantly by the liver [1, 2]. Its serum level may increase to 1000-fold when the body responds to various injuries, including trauma, infection, and inflammation [2]. Besides in the liver, SAA expression and synthesis occur in several tissues and cells, such as synovial tissue, placenta, adipocytes, and smooth muscle cells. SAA is also expressed in diseased tissues and has been found in the atherosclerotic plaques, rheumatoid synovitis, in the brain of patients with Alzheimer, and in tumor cells [3]. 

The SAA gene family, composed by three isoforms (SAA1, SAA2, and SAA4), is upregulated in human tumors [4]. This finding has prompted a number of clinical studies in which a direct correlation between high concentrations of SAA in the serum of cancer patients and their tumor grading has been investigated [4]. The inverse correlation between plasma SAA concentration and patient survival leads to the potential use of SAA as a biomarker to monitor cancer patients and as a valuable tool for postoperative followup [5–8].

Besides its potential as a cancer biomarker, the role of SAA in carcinogenesis and neoplastic diseases is also of great interest [4, 9]. The cytokine-like properties of SAA (most of which are likely due to its powerful and rapid induction of cytokine production) affect the course of inflammation and suggest a role for SAA in tumor progression [10]. Recently, we described a growth factor-like activity for rSAA in fibroblasts [11] and preadipocytes [12]. 

It is also known that rSAA induces the expression of iNOS (inducible-nitric oxide synthase) [13] and primes cells for the production of reactive oxygen species (ROS) [14]. rSAA also activates plasminogen [15] and matrix metalloproteinases (MMPs) [16, 17], interacting with the extracellular matrix (ECM) [18] and activating ECM degradation [17]. Furthermore, it induces chemotaxis [19], cell adhesion, migration [20, 21], proliferation [11, 12], and invasion [16].

Demonstration of a direct activity of rSAA on the invasiveness of tumor cells is limited to a single study that showed the induction of MMP-9 and invasiveness promoted by rSAA in a renal cell carcinoma line [16]. Here, we investigated the effect of rSAA on the expression and activity of MMP-2 and MMP-9 in two human glioma cell lines, A172 and T98G, and the correlation with cell proliferation, migration, and invasion. Gliomas are the most common adult primary brain tumor and are characterized by a highly aggressive behavior and propensity for infiltration and metastasis [22]. Given that the susceptibility to develop a glioma seems to be associated with genetic inflammatory patterns [23], we also investigated the effect of rSAA on the production of molecules involved in inflammation and tumor progression, such as the chemokine IL-8, nitric oxide (NO), and reactive oxygen species (ROS). Expression and production of all SAA isoforms were also analyzed. Our findings support a direct contribution of SAA to tumor development, progression, and metastasis that depends on the cell type and concentration of SAA. 

## 2. Materials and Methods

### 2.1. Cell Culture

 Human glioma A172 and T98G cells lines were acquired from the American Type Culture Collection (Manassas, VA, USA) and maintained in Dulbecco's Modified Eagle's Medium (DMEM) (Gibco), supplemented with 10% fetal bovine serum (FBS) (Sigma) containing 100 IU/mL of penicillin and 100 *μ*g/mL of streptomycin. Cells were cultured at 37°C in a humidified atmosphere at 5% CO_2_.

### 2.2. Proliferation Assay

Gliomas (1 × 10^4^ cells/mL) were plated in 96-well plates. When 70% confluent, the cells were synchronized for 48 h and stimulated with 0.1, 1, 5, and 20 *μ*g/mL of human recombinant SAA1 (rSAA) (PeproTech Inc. Rocky Hill, NJ, USA) for 48 h. Then, 0.5 *μ*Ci/well of [^3^H-methyl]-thymidine (Amersham) was added for 18 h before completing the 48 h of stimulation with rSAA. Cells were fixed with ice-cold 10% trichloroacetic acid (Sigma), lysed with 0.5 M NaOH (Merck), and transferred to Whatman filter papers (1.0–0.5 cm). The filters were washed with 5% trichloroacetic acid, followed by 70% ethanol and finally acetone (Merck). The filters were placed in vials containing 2 mL of scintillation fluid (PPO 4 g, POPOP 0.1 g, and toluene 1L) and radiation was measured using the liquid scintillation counter (Beckman Instruments, Palo Alto, CA, USA).

### 2.3. Migration by Scratch Assay

A172 and T98G cells (5 × 10^4^/well) were plated in 24-well plates. After 24 h, the cells reached confluence and a vertical center line (wound) was opened with the tip of a pipette 200 *μ*L. The cells were treated with 20 *μ*g/mL of rSAA and the migration of cells in the presence of SAA was compared with migration of control cells during 24 h. Images were acquired at zero and 24 h in an inverted optical microscope (Nikon TS100; Japan) at 100x with a 2.4x optical zoom. The migrated area was quantified by AxioVision Release 4.8 software (Carl Zeiss International). 

### 2.4. Invasion Assay

Transwell chambers containing polycarbonate filters (8 *μ*m, Costar Corp.) were coated on the upper surface with matrigel (Becton–Dickinson). Gliomas cells (6 × 10^4^/well) were added to the upper chamber with rSAA (5 and 20 *μ*g/mL) proportional to the total volume of medium in the top and bottom of the Boyden Chamber. After 48 h, the cells that invaded the matrigel and reached the lower surface of the filter were fixed in 5% glutaraldehyde (Sigma) solution, stained with a toluidine blue (Merck) staining solution (0.5%), and ten random fields per filter were counted under a light microscope at 100x magnification with a 2.4x optical zoom.

### 2.5. RNA Extraction and Reverse Transcription

Glioma lines A172 and T98G (2 × 10^5^ cells/mL) were plated and cultured in 35 mm plates for 48 h. Total RNA was extracted from 2 × 10^5^ cells/mL with RNeasy Micro kit (Qiagen, Hilden, Germany) according to manufacturer's instructions. RNA concentration was analyzed on a spectrophotometry (ND1000). A total of 500 ng/*μ*L of RNA, previously treated with DNase I (Invitrogen), was retrotranscribed into cDNA using SuperScript First Strand kit (Invitrogen) according to the manufacturer's instructions.

### 2.6. Quantitative Real-Time PCR

 The following specific primers were used: SAA1 (forward 5′-CCTGGGCTGCAGAAGTGATCAGCGA-3′ and reverse 5′-AGTCCTCCGCACC-ATGGCCAAAGAA-3′) (NM_199161.2), SAA2 (forward 5′-CTGGGCCGCAGAAGTGA-TCAGCA-3′ and reverse 5′-GAGTCCTCCGCACCATGGCCTGT-3′) (NM_030754.3), SAA4 (forward 5′-GTTCGTTTTTCAAGGAGGCTCTCCAA-3′ and reverse 5′-GGATATCATTATGTCCCAATAGGCTCT-3′) (NM_006512.2), MMP-2 (forward 5′-GACTACGACCGCGACAAGA-3′ and reverse 5′-TGTTGCCCAGGAAAGTGAA-3′) (NM_004530.4), MMP-9 (forward 5′-GAGGTGGACCGGATGTTCC-3′ and reverse 5′-AACTCACGCGCCAGTAGAAG-3′) (NM_004994.2), and Tubulin (forward 5′-TCAACACCTTCTTCAGTGAAACG-3′ and reverse 5′-AGTGCCAGTGCGAA-CTTCATC-3′) (NM_006082.2), which was used as a constitutive control. BLAST searches were conducted on all primer sequences to ensure gene specificity. Quantitative RT-PCR was performed in the Gene AMP 7500 Sequence Detection System (PE Applied Biosystems), with the SyBr Green Master mix (Applied Biosystems, Mount Holly, NJ, USA). Each PCR reaction contained 1.0 ng of cDNA, 6 *μ*L SyBr Green master mix, and 3 *μ*L of forward and reverse primers at 600 nM. Reaction conditions were as follows: 95°C for 10 min, 40 cycles of 95°C for 10 s (melting), and 60°C for 1 min (annealing and elongation). The cycle threshold (Ct) for each run was set as 0.1 when amplification was observed in log phase. Relative gene expression was determined using the ΔΔCt method. The efficiency of each reaction was validated as previously described [24].

### 2.7. Measurement of MMP-2 and MMP-9 Activity

 A172 and T98G lines (1 × 10^5^ cells/well) were plated in 24-well plates. When 70% confluent, the cells were synchronized and stimulated with 5 and 20 *μ*g/mL of rSAA for 48 h. MMP-2 and MMP-9 activity was quantified in the culture supernatants from monolayer cultures using specific Biotrak assay systems (MMP-2 Biotrak Activity Assay RPN 2631; MMP-9 Biotrak Activity Assay RPN2634, GE Healthcare, Buckinghamshire, UK) according to the manufacturer's instructions. One hundred micrograms of total protein from the corresponding culture medium were added to each well of the Biotrak plates. The absorbance was measured by spectrophotometry at 405 nm. MMP-2 and MMP-9 activities were calculated using standards provided by the kit and expressed as ng/mg of total protein. Each cell line was analyzed in triplicate.

### 2.8. Cytokine Determination

 A172 and T98G lines (1 × 10^4^ cells/mL) were placed in the culture medium in the presence or absence of recombinant human SAA1 (PeproTech Inc. Rocky Hill, NJ, USA) for 48 h. Cell-free supernatants were collected, centrifuged, and assayed for IL-8 (detection limit = 31.2 pg/mL) by enzyme-linked immunosorbent assay (ELISA) (DuoSet, R&D System, Minneapolis, MN, USA) as described by the manufacturer.

### 2.9. Superoxide Anion Determination

Lucigenin (1 mM) was added to gliomas (1 × 10^4^ cells/mL) incubation medium. Immediately afterwards, cells were treated with 5 and 20 *μ*g/mL of rSAA. ROS release was monitored for 3 h in a Microplate Luminometer (EG&G Berthold LB96V, New Haven, CT, USA). The assays were run in PBS buffer supplemented with CaCl_2_ (1 mM), MgCl_2_ (1.5 mM), and glucose (10 mM) at 37°C [11]. The results were expressed as relative luminescence units (RLUs).

### 2.10. Chemiluminescence Assay for the Detection of NO Products

Determination of NO products in the A172 and T98G cell lines was performed with a chemiluminescence NO analyzer (NOA^280^; Sievers Instruments) following optimization. A172 and T98G lines (1 × 10^4^ cells/mL) were plated in culture medium on 24-well plates in the presence or absence of rSAA (0.1, 1, 5, and 20 *μ*g/mL) for 48 h. Cell-free supernatants were collected and 60 *μ*L of supernatant were injected into a vessel containing a solution of 45 mM potassium iodide and 10 mM iodine in glacial acetic acid maintained at 60°C continuously purged with nitrogen. Under these conditions, NO products were reduced to NO gas, which is carried on the stream of nitrogen into the chemiluminescence detector. The peak areas of the samples were calculated with the instrument software and compared with those of standard solutions of nitrate and nitrite analyzed under the same experimental conditions [25].

### 2.11. SAA Determination

 A172 and T98G cells (1 × 10^5^ and 5 × 10^5^/mL) were plated on 35 mm plates (Corning Incorporated). After 48 h, cells were washed with PBS, trypsinized, lysed with Tween 0.05% (Merck) by sonication (Branson Ultrasonics Corporation, Danbury, CT, USA) for 20 s at 30 W and centrifuged. The supernatants were analyzed using a human SAA ELISA kit (Invitrogen, Camarillo, CA, USA).

### 2.12. Statistical Analysis

 Results are shown as the means ± SEM of the three experiments in triplicate. Statistical analyses were performed with Graph Pad Prism5 (Graph Pad Software Inc., San Diego, CA, USA). Multiple samples were compared with one independent variable, one-way analyses of variance (ANOVA) followed by Student-Newman-Keuls post hoc test. Student's *t*-test was also performed when comparing nonstimulated and stimulated conditions for independent experiments. The level of significance was set at *P* < 0.05. 

## 3. Results

### 3.1. rSAA Induces Cell Proliferation

**Figure 1:**
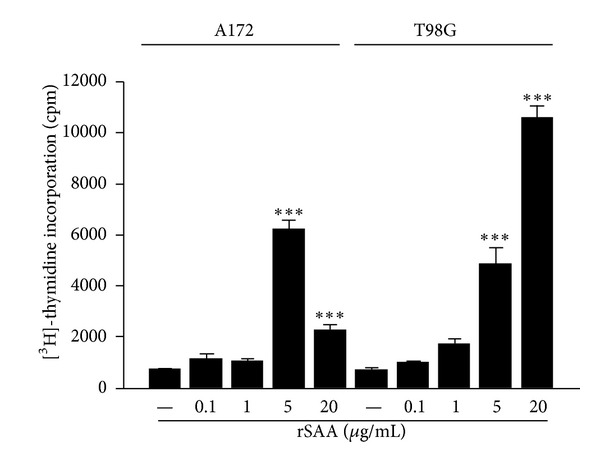
rSAA induces cellular proliferation in glioma cell lines. A172 and T98G cells were treated with increasing concentrations of rSAA (0.1–20 *μ*g/mL) for 48 h. During treatment, the cells were labeled with [^3^H-methyl]-thymidine. An increase in the incorporation of [^3^H-methyl]-thymidine can be observed in the two cell lines when they are treated with higher concentrations of rSAA, showing an increase in cellular proliferation of gliomas. Data represent mean ± SEM of three independent experiments. ***P < 0.001 versus control.

rSAA increased proliferation of A172 and T98G cells, according to the assay of [^3^H]-thymidine incorporation. Cells grew to 70% confluence and then were deprived for 48 h in medium with 0.5% FBS and then stimulated with rSAA (0.1 to 20 *µ*g/mL) for 48 h. For the A172 line the proliferative effect of rSAA was not dose-dependent and the optimum concentration of rSAA to induce A172 proliferation was around 5 *µ*g/mL whereas treatment of T98G cells with rSAA promoted a clear dose-dependent effect on cell proliferation (Figure 1). 

### 3.2. rSAA Affects Cell Migration and Invasion and MMPs Activity

**Figure 2:**
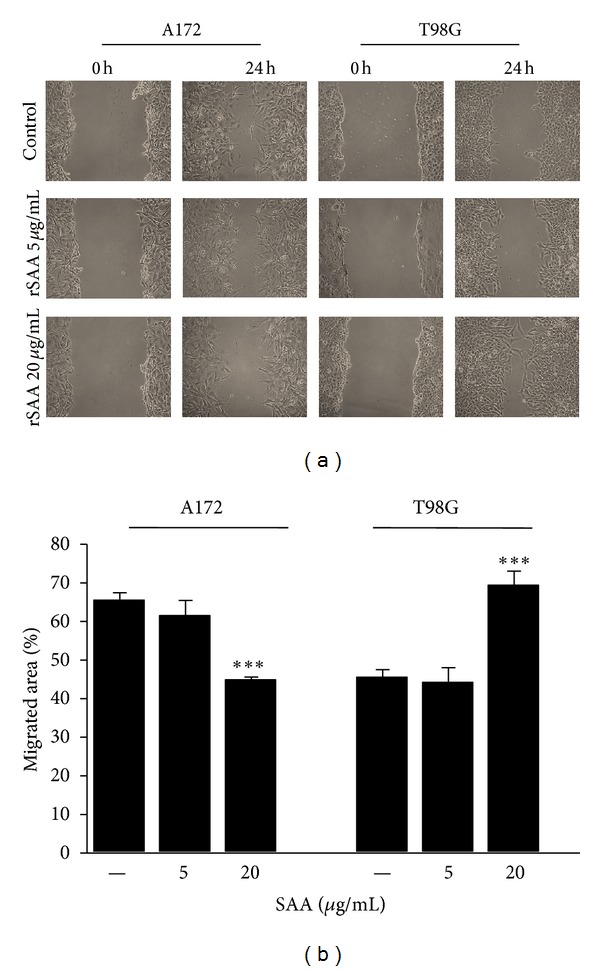
rSAA affects migration of human glioma cell lines. (a) Scratch wound in the presence of rSAA for 24 h showed that SAA decreased migration of A172 and increased migration of T98G cells when stimulated with 20 *μ*g/mL rSAA. Photos are representative of three experiments. (b) The percent in the migrated area of the scratch region was quantified and is presented in the graphic. Data represent mean ± SEM of three independent experiments. ***P < 0.001 versus control.

**Figure 3:**
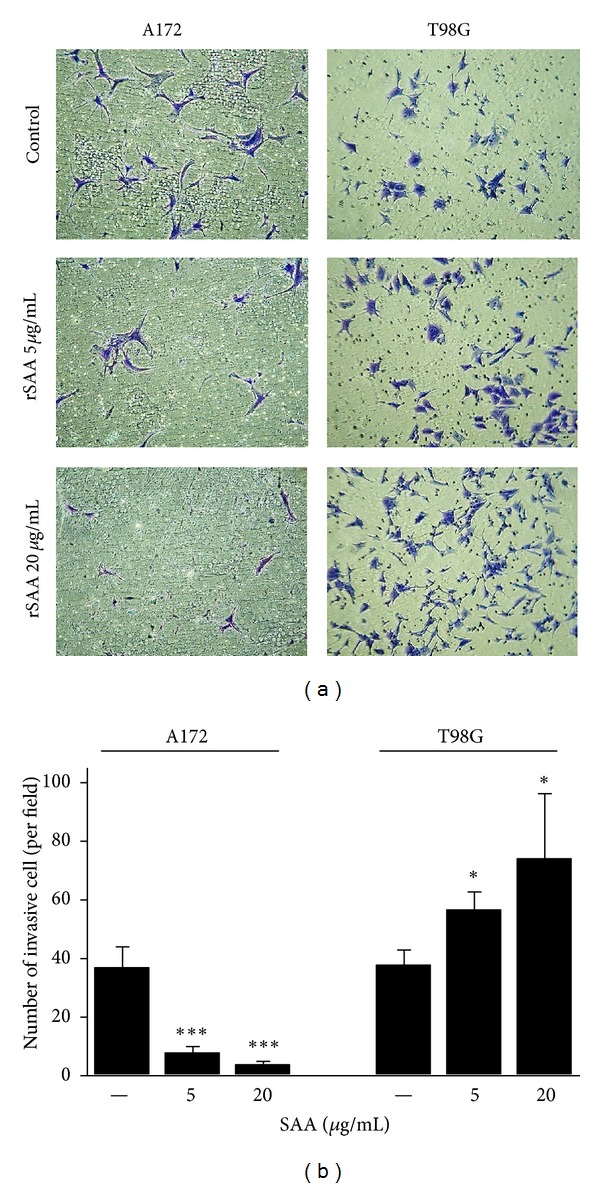
rSAA (5 and 20 *μ*g/mL) affects invasion of human glioma cell lines. (a) The invasion of A172 cells decreased in the matrigel in the presence of rSAA while the invasion of T98G cells increased after 48 h. Photos are representative of three independent experiments. (b) Quantitative representation. *P < 0.05,^  ∗∗∗^ *P* < 0.001 versus control.

rSAA differently affected migration and invasion in the two glioma cell lines. Notably, rSAA stimulated cell movement and was chemotactic for T98G cells. The scratch assay showed that after 24 h there was an increase in motility, with T98G migrating to occupy an area 50% larger than the area previously occupied, when 20 *µ*g/mL rSAA was present (Figures 2(a) and 2(b)). In contrast, the treatment with rSAA resulted in an inhibition in which the area occupied was reduced in 30%, in the A172 cell line. In the matrigel transwell assay the effect of rSAA was dose dependent. Invasion of T98G to the bottom chamber after 48 h was almost twice greater when 20 *µ*g/mL rSAA was present (Figures 3(a) and 3(b)). In the A172 cell line, however, rSAA inhibited invasion by approximately 90%.

**Figure 4:**
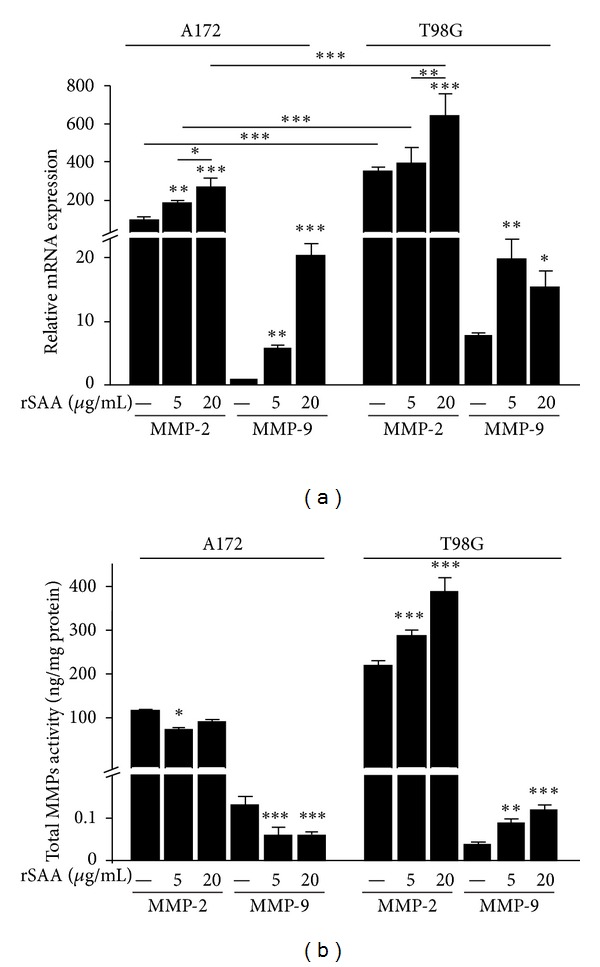
rSAA affects mRNA expression and gelatinolytic activity of MMP-2 and -9 in A172 and T98G cells. (a) Quantitative real-time PCR was performed to assess the mRNA expression of MMP-2 and -9 on gliomas when stimulated with rSAA (5 and 20 *μ*g/mL) for 48 h. The mRNA expression of MMP-2 and MMP-9 is increased by rSAA treatment in both gliomas. (b) Enzymatic activity of MMPs was measured in cellular supernatant. Activity data showed a decrease in MMP-2 and MMP-9 activity in A172 cell line, and an increase in MMP-2 and MMP-9 activity in T98G cell line. Data represent mean ± SEM of three experiments. *P < 0.05, **P < 0.01, and ***P < 0.001 versus control.

The migration and invasion processes require extensive remodeling of the extracellular matrix by MMPs [26]. Here, we show that the constitutive MMP-2 expression and activity were higher than that of MMP-9 for both glioma cell lines (Figures 4(a) and 4(b)). In addition, the T98G cell line constitutively expressed more mRNA for these MMPs than did A172. The basal and SAA-increased levels of MMP-2 and MMP-9 expression for both glioma lines are shown in Figure 4(a). rSAA significantly increased mRNA expression of these two metalloproteinases. On the other hand, when the gelatinolytic activity of the MMPs was evaluated, we observed that rSAA inhibited the activity of MMP-2 and -9 enzymes in A172 while it increased these activities in T98G (Figure 4(b)). The results from gelatinolytic activity confirmed the results from the migration and invasion assays for both lines (for comparison see Figures 2 and 3).

### 3.3. rSAA Affects IL-8, Induces ROS and NO in Human Glioma Lines

**Figure 5:**
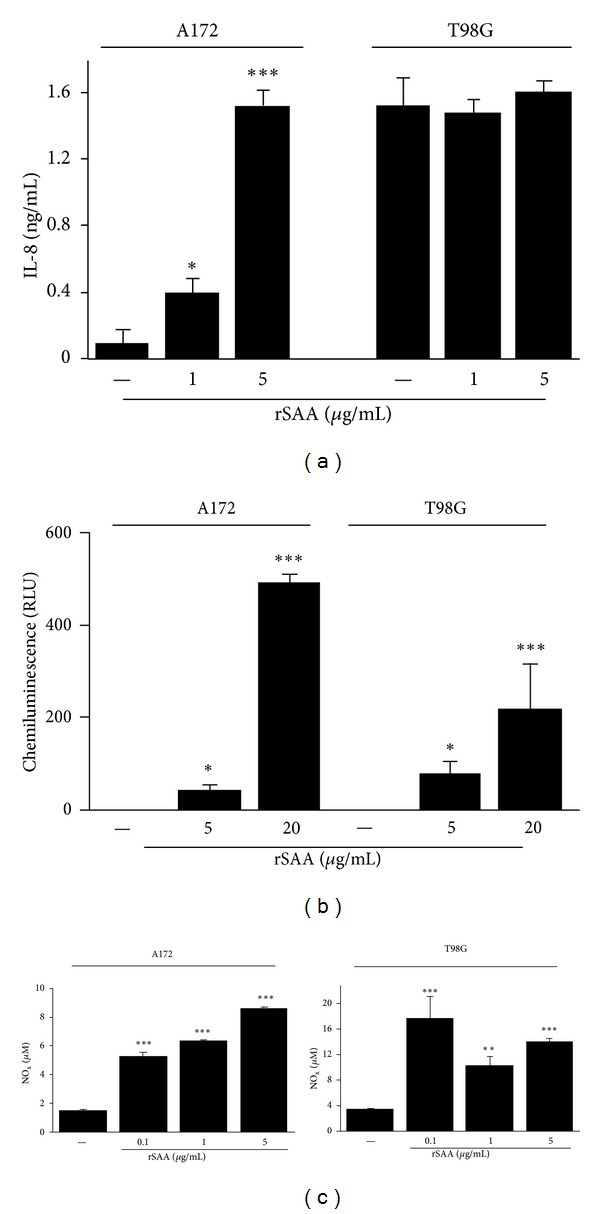
rSAA stimulates the production of relevant molecules in the tumor microenvironment. (a) rSAA (1 and 5 *μ*g/mL) stimulates the production of IL-8 in a dose-dependent manner by A172 but not by T98G cells. (b) Dose dependence effect of rSAA (5 and 20 *μ*g/mL) on O_2_^−^ anion production as measured by lucigenin-enhanced chemiluminescence in gliomas. (c) SAA stimulates the production of NO in glioma cell lines even in lower concentrations. Data are mean ± SEM of three independent experiments. *P < 0.05, **P < 0.01, and ***P < 0.001 versus control. NO_*x*_—nitric oxide products.

Many tumors secrete cytokines [27] and produce ROS [28] and NO [29]. In this study, we show that the A172 glioma did not produce IL-8 in nonstimulated conditions. However, when these cells were treated with rSAA, the production of IL-8 was higher and dose dependent (Figure 5(a)). On the other hand, the production of IL-8 remained as in the baseline and unaffected by the addition of rSAA in the T98G line.

To test if rSAA was able to trigger ROS production in glioma cells, we used the lucigenin-amplified chemiluminescence assay that was previously shown to be a sensitive test to measure the rise in O_2_^−^ levels [11]. While in nonstimulated conditions gliomas did not produce ROS, the incubation of glioma cells with rSAA showed a positive correlation between rSAA concentration and O_2_^−^ production (Figure 5(b)). 

It is known that SAA can trigger iNOS expression and NO production in macrophages [30], thus we investigated the possibility of this effect also occurring in tumor cells. SAA triggered NO production in a dose-dependent way in A172 cells, even at 0.1 *μ*g/mL SAA (Figure 5(c)). SAA did affect NO production in the T98G line but this effect was not dosedependent. 

### 3.4. SAA Is Expressed and Produced in Glioma Cell Lines

**Figure 6:**
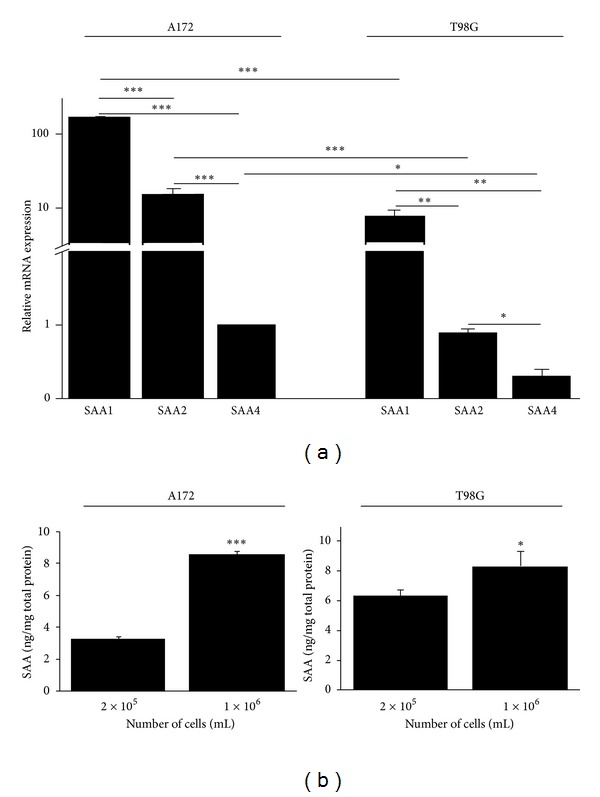
SAA is expressed and produced in A172 and T98G cells. (a) Quantitative real-time PCR was performed to assess the mRNA expression of SAA isoforms SAA1, SAA2, and SAA4, in A172 and T98G cells. (b) The production of SAA protein was measured in two cell densities by ELISA. Data represent the mean ± SEM of three experiments. *P < 0.05, **P < 0.01, and ***P < 0.001.

Because SAA is an acute-phase protein predominantly expressed and produced in the liver in response to inflammation, we tested whether these glioma cells express and produce the isoforms SAA1, SAA2, and SAA4 in basal conditions. Both lines expressed all the isoforms of SAA (Figure 6(a)). However, the relative expression level of SAA isoforms in A172 cells was higher than in T98G cells. The level of SAA1 expression was 10 times higher than SAA2 and 100 times higher than SAA4. The production of SAA protein was higher in T98G cells compared to A172 when the density of 2 × 10^5^ cells /mL was tested and was similar to density of 1 × 10^6^ cells /mL (Figure 6(b)). 

## 4. Discussion

In this study, we have shown that SAA induced proliferation and affected migration and invasiveness of two human glioma cell lines. SAA also affected the production of important mediators associated to tumor growth, such as IL-8, ROS, NO, and MMPs. We have also shown that these cell lines constitutively express and produce SAA. These findings show that SAA may have a role in tumor progression and metastasis. The involvement of SAA in tumor progression has been predicted on the basis of its effect on the modulation of cytokines [31–33] and MMPs production [16, 20]. Nevertheless, here we describe a dual effect of SAA on the activity of MMPs that may contribute to the invasive behavior of two glioma cell lines.

SAA affected glioma proliferation in a growth factor-like manner. SAA produced a marked effect on cell proliferation of T98G cells, while a more modest and not dose-dependent effect was observed on A172. In each cell line, SAA affected cell migration and invasion differently and showed entirely opposite effects (Figures 2 and 3), most likely as the result of its differential modulation of MMP activities. rSAA increased the mRNA expression of MMP-2 and -9 in gliomas (Figure 4(a)) while it presented a dual effect on the activity of these enzymes (Figure 4(b)). MMPs are essential for proper ECM remodeling and invasion [34] while MMP-2 and MMP-9 are known to be overexpressed in gliomas [35]. The interaction of rSAA with the extracellular matrix, involving MMPs, has been previously described in monocytic cells [36, 37] but has not been extensively described in tumor cells [16]. We showed here that, depending on the cell type, SAA can inhibit or activate MMPs. 

The A172 and T98G glioma lines represent models of human carcinomas characterized by high aggressiveness. Gliomas exhibit numerous mutations in genes that control cell cycle and induce proliferation and migration [38]. They also exhibit different profiles regarding the expression and production of growth factors and cell–cell adhesion molecules. Some examples are Tp53 [39], PTEN [40], VEGF [41], and ADAM23 [42]. These genetic differences are probably the basis for the different results between A172 and T98G observed in this study in response to SAA and may also explain the findings obtained in a previous study in which the effect of IGF-1 was tested in these two cell lines [43]. 

IL-8 represents one of the many factors that influence tumor growth [44–46]. We found a remarkable difference between A172 and T98G glioma lines regarding the production of IL-8 (Figure 5(a)). In previous studies, it has been shown that SAA induced IL-8 production by human leukocytes [31, 47] and human epithelial colorectal adenocarcinoma cells [48]. Here we observed that although A172 cells did not produce IL-8 in basal conditions, they responded to SAA in a dose-dependent way. In contrast, T98G spontaneously produced a relatively high amount of IL-8. Nevertheless, the cell line was not responsive to SAA. 

Significant differences between the two cell types were also found for NO. NO and other metabolites are involved in genotoxicity and carcinogenesis, and NO may correlate with tumor grade and metastasis. Expression of iNOS has been reported in many tumor tissues [49]. We found that SAA induced NO in both tumor lines but in a different manner (Figure 5(c)). Whereas the A172 line responded to SAA in a dose-dependent manner, the same was not observed for T98G. Differences of the same nature were also found by exposure of these two lines to IFN-*γ*, TNF-*α*, IL-1*β*, and lipopolysaccharide (LPS) [50].

As previously described for fibroblasts, there is strong evidence that ROS production and cell proliferation induced by SAA are correlated events [11]. Activation of growth factor-stimulated signaling cascades by low levels of ROS results in increased cell cycle progression [51]. In 3T3 fibroblasts the ROS production in response to SAA seems to occur, at least in part, due to activation of the NADPH oxidase enzymes. It is reasonable to assume that in the glioma cell lines studied here the increments in superoxide anion resulted from regulation of the intracellular redox state by SAA, with an important impact on redox-sensitive signaling cascades acting on cell cycle. 

Signs of chronic inflammation are present in virtually all tumors [27, 52, 53]. It has been shown that glioma patients have elevated levels of inflammation serum markers such as IL-6, TNF-*α*, and reactive protein C [54]. Also, angiogenesis in gliomas is triggered by a number of proteins secreted by tumor cells, and by stromal and inflammatory cells [55]. In this study, we identified SAA as an additional inflammatory factor that may link inflammation with tumor progression. We also show that two glioma cell lines express and produce SAA and that expression may be cell-type dependent.

**Figure 7:**
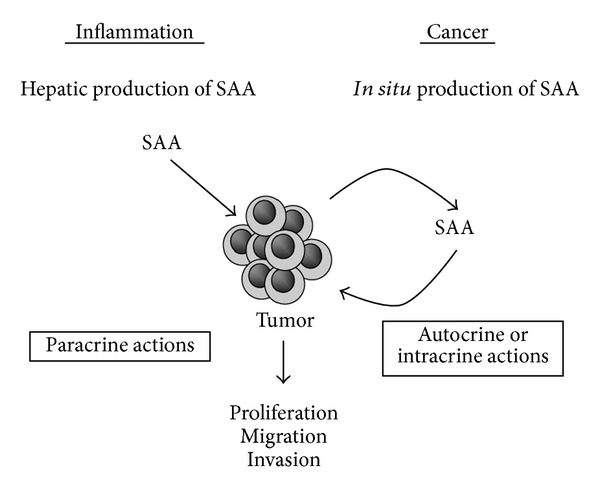
Influence of SAA on tumor cells. Glioma cells may be affected by SAA produced by the liver during inflammatory processes (paracrine actions) and by SAA synthesized by the tumor itself (autocrine or intracrine actions). The effect on glioma cell line proliferation, migration, and invasion depends on cell type and concentration of rSAA.

Autocrine, intracrine, and paracrine factors able to induce proliferation, migration, and invasion have been the focus of research projects that seek to understand the basis for the invasive capacity of tumors, including gliomas [56]. SAA synthesized by the liver, in acute and chronic conditions, may exert paracrine effects on tumor cells. Our results support the hypothesis that autocrine and intracrine actions of SAA expressed in glioma cells are also possible. SAA produced by the liver during inflammation and the SAA produced by the tumor itself may affect tumor cell biology (Figure 7). 

Future studies should invest efforts in identifying SAA-receptors and the signaling pathways triggered by SAA in gliomas, and in investigating the effects of SAA on the cytoskeleton major protein and on invasiveness using more complex assays, such as the invasion assay in brain slice model [57]. 

Our results support the hypothesis that SAA is not only a liver-secreted protein, but also a tumor cell product that may have an important role in tumor development and progression, suggesting a novel role for SAA in tumorigenesis.
